# Iranian nurses’ perceptions of core competencies required for disaster risk management

**DOI:** 10.1186/s12873-023-00853-3

**Published:** 2023-08-04

**Authors:** Faezeh Soltani Goki, Hojjat Farahmandnia, Amirreza Sabzi, Gulcan Taskiran Eskici, Jamileh Farokhzadian

**Affiliations:** 1 Student Research Committee, Razi Faculty of Nursing and Midwifery, Kerman University of Medical Sciences, Kerman, Iran; 2 Health in Disasters and Emergencies Research Center, Institute for Futures Studies in Health, Kerman University of Medical Sciences, Kerman, Iran; 3 School of Medicine, Shiraz University of Medical Sciences, Shiraz, Iran; 4 Department of Nursing Administration, Faculty of Health Sciences, Ondokuz Mayis University, Samsun, Turkey; 5 Nursing Research Center, Kerman University of Medical Sciences, Kerman, Iran

**Keywords:** Nurses, Disaster risk management, Perception, Disaster competencies, Hospital

## Abstract

**Background:**

With an increase in the incidence and severity of disasters, disaster risk management receives an important priority in the health system. Nurses at all levels of healthcare play an important role in disaster risk management and they must have core preparation and competencies to respond to disasters. This study aimed to evaluate Iranian nurses’ core competencies required for disaster risk management.

**Methods:**

This cross-sectional study was conducted on 277 nurses working in three hospitals affiliated to Kerman University of Medical Sciences in 2022. The demographic information questionnaire and the nurses’ perceptions of disaster core competencies scale (NPDCC) were used to collect data, and SPSS21 was used to analyze data.

**Results:**

Nurses’ competencies in disaster risk management were favorable (3.67 ± 0.77), with the subscales of technical skills and special diagnostic skills receiving the highest (3.91 ± 0.65) and the lowest mean scores, respectively (3.46 ± 0.87). The results found a significant difference between the scores of nurses’ competencies in disaster risk management in terms of education level, age, work experience, employment status, participation in training courses related to disaster risk management, work experience in incidents and disasters.

**Conclusion:**

The study results suggested nurses’ high level of competency in disaster risk management, but indicated gaps in their core competencies. The study results recommend authorities provide various training courses related to disaster risk management for all nurses.

## Introduction

Statistics indicate that disasters are increasing and it is impossible to predict and prevent the occurrence of disasters [1], and with an increase in natural and manmade disasters, they still have a negative impact on human (physical, mental, psychological, social, economic), capital, or the environment [2]. In the last two decades, disasters have directly affected the lives of approximately 1.23 million people [3]; Asia is the most affected region in the world by all types of disasters, as 44% of all disasters, 58% of all deaths, and 70% of all victims happen here [4]. Iran is always one of the most affected countries by various types of disasters [5].

The working conditions of healthcare providers, including nurses, change during emergencies and disasters. Inconsistencies, lack of facilities and resources, provision of care for diverse and chronically ill patients, numerous inter- and extra-organizational communications, increased workload, physical and psychological pressures create a different situation for nurses [6]. Countries all over the world have already planned and taken measures to manage disaster risk properly [7].Disaster risk management is to reduce disaster effects on health, facilitate the use of healthcare resources, improve preparedness and the ability to respond in time, and recover after disasters [8]. The health system plays the most important role during emergencies and disasters because it must fulfill health-related needs for people in such crises [9]. Nurses are the largest group involved in disaster risk management, so they must develop their competencies in disaster risk management [10].

Disaster risk management competency for nurses refers to providing nursing care effectively to disaster victims using the necessary knowledge and skills [11].Disaster nursing education and training programs had increased in the world over the past 20 years. Because nursing leaders and educators alerted of the necessity to offer education and training for nursing students and nurses [12]. In Iran, the undergraduate nursing curriculum included 1.5 credits of nursing in accidents and disasters (34 h, in the third semester-1theoretical credits and 0.5 practical credits). The postgraduate nursing curriculum included 2 credits of basics of disaster management (34 h, in the third semester-2 theoretical credits). Also, they participate in irregular training courses such as training sessions, maneuvers, round table maneuvers after entering the clinical environment [13].

Regarding the nature of their practice and the role they have in treatment, rehabilitation and control, nurses’ competencies in disaster response are important to reduce the negative consequences of disasters on the health of affected people [14]. Nurses’ competencies in disaster risk management create a strong sense of readiness to help disaster victims [6, 15] and reduce their anxiety and hesitation [16]. Competent nurses can provide quality care and psychological support to affected clients, increase public trust in healthcare providers, and reduce mortality and complications after disasters [17] and it is essential to assess nurses’ competencies in disaster risk management in different environments and levels of healthcare; these evaluations make managers aware of nurses’ competencies in disaster risk management and eliminate their shortcomings in this area [18] therefore, evaluating and improving their competencies can be a source of changes and preparations in the health system [19].

Recent studies in the Europe, US, Australia, China, and Turkey investigated nurses’ roles, preparations, perceptions, knowledge, psychological characteristics, and competencies in disaster risk management and reported that nurses had shortcomings at least in one of the subscales of the nurses’ perceptions of disaster core competencies scale. The studies emphasized that nurses were confused and shocked during incidents and disasters as they were unprepared; therefore, these studies highlighted the need to improve their competencies [20–24]. Other studies suggest that the assessment of nurses’ core competencies in disaster risk management explains the need to include courses related to disaster management in nurses’ curricula and helps plan continuous and in-service training courses, disaster exercises and maneuvers to meet the needs of nurses [15, 25]. However, there is a lack of evaluation on competencies in disaster risk management for nurses to determine the gaps in curriculum development and continuing education to prepare nursing students and nurses with the knowledge and skills required for disaster risk management [12]. Limited studies in Iran emphasized the assessment of nurses’ competencies in different situations [26, 27]. Identifying and examining nurses’ core competencies in disaster risk management can provide valuable information for policymakers and planners, so this study aimed to evaluate Iranian nurses’ core competencies required for disaster risk management.

## Method

### Study design and setting

This cross-sectional study was conducted in three hospitals of Bahonar, Shafa and Afzalipur affiliated to Kerman University of Medical Sciences from April to June 2022.

### Study population and sampling

The study population consisted of all nurses (N = 1600) working in the hospitals affiliated to Kerman University of Medical Sciences at the time of data collection. The sample size was calculated to be 309 using Cochran’s formula (α = 0.05, d = 0.05, Z = 1.96). Stratified random sampling was used to select nurses, and 103 nurses were randomly selected from each hospital. The inclusion criteria included a bachelor’s or higher degree in nursing and at least one year of work experience. Finally, 277 nurses completed the survey (response rate = 89.6).

### Data collection tool

Two questionnaires were used to collect data:

Demographic characteristics questionnaire: This questionnaire collected information about age, gender, marital status, employment status, professional experience, educational degree, work position, work experience in prior disaster, and attending in the disaster risk management training course.

The nurses’ perceptions of disaster core competencies scale (NPDCC): Celik (2010) developed, accredited and psychometrically measured this scale in Turkey to investigate nurses’ perceptions of core competencies during disasters. This 45-item scale includes five subscales: critical thinking skills (4 items), special diagnostic skills (6 items), general diagnostic skills (13 items), technical skills (14 items), and communication skills (8 items). The scale is Likert-type, where each item is scored from one (this needs to be taught) to five (I can do it and teach it), with the lowest and highest scores being 1 and 5, respectively. A higher score indicates nurses’ better perceptions of core competencies [28]. A score of 1-2.33 was considered as poor competency, a score of 2.34–3.66 was considered as moderate competency, and a score above 3.66 was considered as high competency [28, 29]. Taskiran et al. confirmed the reliability of original NPDCC on Turkish nurses using internal consistency and reported Cronbach’s alpha coefficients of 0.81–0.92 and 0.96 for the subscales and the whole scale, respectively [29]. In order to use the NPDCC in this study, permission was obtained, and translation-back translation was used for cultural adaptation of its Persian version. The research team translated the NPDCC into Persian, two English language experts translated it back into English, and then the inconsistencies were checked, and the Persian version of the questionnaire was prepared after changes in phrases. To determine the qualitative content validity, the NPDCC was given to 10 faculty members of the School of Nursing and Midwifery and Health in Disasters and Emergencies Research Center, and we prepared the final scale after collecting their opinions. This scale was given to 20 nurses to check the perceptions of the research participants; no changes were made in the scale. The NPDCC reliability was determined using a pilot study on 30 nurses. Cronbach’s alpha coefficients for the subscales of critical thinking skills, special diagnostic skills, general diagnostic skills, technical skills, communication skills, and the whole scale were 0.86, 0.86, 0.94, 0.94, 0.96, and 0.96, respectively.

### Data analysis

SPSS21, descriptive (mean, standard deviation and percentage) and inferential statistics (independent t-test, one-way analysis of variance, Tukey’s post hoc test) were used to analyze data. According to the test results, the Kolmogorov-Smirnov test was used to check the data normality. A significance level less than 5% was considered.

## Results

The results indicated that 277 nurses completed the survey (response rate = 89.6). Most of the participants were female (81.6%), married (76.9%), clinical nurses (43.3%) and aged above 40 years old (40.8%). They had a bachelor’s degree (80.5%), a work experience of 11–20 years (51.6%), were permanent staff (65%) and 53.4% of them participated in training courses related to incidents and disasters, and 43.7% had work experience in previous incidents and disasters, such as earthquakes or road accidents, showed in Table 1.

**Table 1:** Demographic characteristics of nurses and the difference in the Score of disaster management competencies according to demographic characteristics (n = 277)

Variables	Categories	n	%	Mean ± SD	Statistic test	P-value
Age	≤ 30	60	21.7	161.5 ± 26.8	F = 1.7	0.2
	31–40	104	37.5	169.2 ± 29.2		
	≥ 40	113	40.8	167.9 ± 24.9		
Gender	Female	226	81.6	172.9 ± 30.5	t=-1.7	0.09
	Male	51	18.4	165.7 ± 26.2		
Educational degree	Bachelor’s degree	223	80.5	165.5 ± 27	t = 4.2	0.01*
	Master’s degree	54	19.5	186.8 ± 23.2		
Marital status	Married	213	76.9	166.8 ± 26.7	t = 0.03	0.9
	Single	64	23.1	167.7 ± 28.5		
Employment status	permanent staff	180	65	170.8 ± 26.8	t = 0.27	0.001*
	Not on the permanent staff	97	35	158.8 ± 25.8		
Professional experience (years)	1–5	55	20.3	148.7 ± 23.7	F = 3.7	0.006*
	6–10	30	10.8	173.6 ± 28.6		
	11–20	143	51.6	166.3 ± 27.4		
	≥ 20	49	17.7	171.4 ± 23.4		
Work position	clinical nurses	120	43.3	158.84 ± 25.78	F = 1.4	0.9
	Intensive care unit nurses	75	27.1	165.3 ± 25.9		
	Nurse managers	16	5.8	176.1 ± 23.4		
	Emergency nurses	66	23.8	170.6 ± 27.5		
Work experience in prior disaster	Yes	121	43.7	171.9 ± 27.7	t = 2.7	**0.007**
	No	156	56.3	163.2 ± 26.1		
Attending in the disaster risk management training course	Yes	148	53.4	174 ± 26.3	t = 4.7	**0.001**
	No	129	46.6	159.1 ± 25.9		

*Bold p-values are significant at the level of ≤ 0.05

The total mean score of nurses’ competencies in disaster risk management was high (3.67 ± 0.77), showed in Table 2. The results showed that 53.4% ​​of the nurses had high competencies in disaster risk management, 45.5% had moderate competencies, and 1.1% had poor competencies. The mean scores of subscales were in critical thinking skills (3.73 ± 0.84), special diagnostic skills (3.46 ± 0.87), general diagnostic skills (3.7 ± 0.64), technical skills (3.91 ± 0.65) and communication skills (3.55 ± 0.86). The results showed subscales of technical skills (3.91 ± 0.65) and special diagnostic skills (3.46 ± 0.87) received the highest and lowest mean scores, respectively.

**Table 2:** Score of nurses’ perceptions of core competencies required for disaster risk management

Sub scales	Items	M ± SD Pre item	M ± SD pre subscale
Critical thinking skills	1. I can use the ethical principles and the nationally approved information in order to decide the actions to be taken and prioritize them in case of a disaster.	3.8 ± 0.8	3.73 ± 0.84
	2. During and after the mass casualty, I can make decisions to assess the nursing care needs of the victims.	3.89 ± 0.8	
	3. I can explain the basic nursing care for the individuals, families, society and special groups (children, elders, disabled people and Pregnant etc.) in accordance with the needs of pre-disaster, disaster and post-disaster period.	3.64 ± 0.86	
	4. I can explain the principles of triage applied and accepted in the mass casualties (i.e. START-Simple Triage and Rapid Treatment)	3.6 ± 0.9	
Special diagnostic skills	5. In the case of a disaster, I can assess the risk situations that can affect the health of mine, my team and the victims, together with the disaster response team	3.69 ± 0.81	3.46 ± 0.87
	6. I can recognize the possible indications of the situation the group of people, with the same symptoms, is exposed to.	3.58 ± 0.81	
	7. I can explain the general symptoms and findings of the exposure to the chemical, biological, radioactive, nuclear and explosive substances that threaten human health.	3.2 ± 0.93	
	8. I can update my knowledge on the chemical, biological, radiological, nuclear and explosive substances in accordance with the up-to-date information.	3.47 ± 0.91	
	9. I can explain the essential elements (nature, size, limits, duration etc. of the event) required for the assessment of a mass casualty.	3.23 ± 0.91	
	10. I can determine the groups that may highly likely be affected and require special care (children, elders, people with a suppressed immune system etc.) during the mass casualty.	3.59 ± 0.85	
General diagnostic skills	11. I can get a history of health to assess exposure to chemical, biological, radiological, nuclear and explosive substances.	3.47 ± 0.91	3.7 ± 0.64
	12. I can assess the airway patency and respiration.	3.95 ± 0.76	
	13. I can perform a cardiovascular assessment including the monitoring of the vital signs and the shock signs.	4.01 ± 0.79	
	14. I can assess the dermatological conditions, especially like injury, burn and eruption.	3.86 ± 0.79	
	15. I can do pain assessment.	3.92 ± 0.76	
	16. I can assess the condition of injury from head to foot.	3.81 ± 0.87	
	17. I can do a general gastrointestinal system assessment including stool sampling.	3.52 ± 0.92	
	18. I can do basic neurological assessment.	3.58 ± 0.87	
	19. I can do a basic musculoskeletal system assessment.	3.68 ± 0.86	
	20. I can do a basic mental, spiritual and emotional state assessment.	3.69 ± 0.87	
	21. I can assess the immediate and late psychological reactions/responses of the individual, family and community following mass casualty.	3.44 ± 0.9	
	22. I can refer the victims to the appropriate sources (psychiatrists, Psychologists, consultants, and psychiatric nurses etc.) in order to provide psychological support in the disasters.	3.71 ± 0.82	
	23. I can explain the psychological effects of the disaster on the professional disaster response teams (healthcare professionals, Firefighters, ambulance personnel, police etc.).	3.51 ± 0.9	
Technical skills	24. I can ensure safe drug management (especially vasoactive and analgesic drugs, oral, subcutaneous, intramuscular and intravenous drug administrations, etc.)	3.79 ± 0.84	3.91 ± 0.65
	25. I can provide safe vaccinations for the protection of the community health in disasters.	3.8 ± 0.85	
	26. I know and apply the appropriate nursing interventions against the side effects of the drugs administered.	3.66 ± 0.85	
	27. I can apply basic first aid practices.	3.97 ± 0.76	
	28. I can administer oxygen and apply breathing techniques	4.13 ± 0.78	
	29. I can insert a urinary catheter.	4.14 ± 0.9	
	30. I can insert a nasogastric tube.	4.11 ± 0.93	
	31. I can apply lavage (e.g. eye and wound lavage).	4.03 ± 0.82	
	32. I can perform the basic wound care.	4.03 ± 0.82	
	33. In case of exposure to the chemical, biological, radiological, nuclear and explosive substances, I can start the appropriate isolation and decontamination processes by assessing the needs of the victims, mine and the disaster response team.	3.48 ± 0.91	
	34. I know and can apply the safety concerns and the use of the personal protective equipment.	3.91 ± 0.88	
	35. I can choose and use the personal protective equipment as required.	4.01 ± 0.8	
	36. Taking into account the nature of the exposure factors and/or injuries, I can apply fluid/nutritional therapy in accordance with the medical treatment and follow up the fluid that the patients take in and out.	3.90 ± 0.77	
	37. I can assess the transfer status of the injured individual and perform preparation, care, and follow-up in such a way to ensure the safety of the patient during the transfer.	3.78 ± 0.87	
Communication skills	38. I know the disaster management system of the institution I work for and I can explain my professional role in the emergency plans.	3.51 ± 0.9	3.55 ± 0.86
	39. I can explain the emergency plans at my workplace and the functions of these plans at community, regional and provincial levels.	3.44 ± 0.9	
	40. I know and can apply the importance of the security and privacy issues during the intervention of mass casualties.	3.64 ± 0.83	
	41. I can ensure the appropriate recording of nursing assessments, interventions and care results during and after the mass casualty.	3.62 ± 0.86	
	42. I can refer applications from patients, the media and other sources to appropriate sources for information about mass casualties.	3.55 ± 0.88	
	43. I can explain the basic principles of risk communication to be applied for the individuals and groups affected by disaster during a mass casualty.	3.48 ± 0.85	
	44. I can recognize the fear, panic and stress reactions that the victims, families and disaster response teams can display during a disaster.	3.61 ± 0.84	
	45. I can explain the appropriate coping strategies to provide support for myself and others against negative effects of disasters.	3.57 ± 0.85	
Total Score of NPDCC		3.67 ± 0.77	

Independent-t test indicated that nurses with master’s degree (t = 4.2, p = 0.01), Attending in the disaster risk management training course (t = 4.7, p = 0.01), and work experience in previous incidents and disasters, such as earthquakes or road accidents (t = 2.7, p = 0.007) had significantly higher scores for competencies in disaster risk management. One-way analysis of variance demonstrated a significant difference in the risk management competency score according to professional experience (F = 3.7, p = 0.006), and employment status (t = 0.27, p = 0.001). Tukey’s test indicated that nurses with 1–5 years of work experience were less competent in disaster risk management than other groups, showed in Table 1.

## Discussion

The study results suggested that the total score of nurses’ competencies in disaster risk management was higher than average, and more than half of them reported a high level of competency in disaster risk management. Studies in Australia and the Europe also reported that nurses’ competencies in disaster risk management were above average [6, 9], but a systematic review study found that nurses had a low to moderate level of preparedness in disaster risk management in Saudi Arabia, Jordan, Indonesia and Pakistan, and less than a quarter of them had disaster core competencies [3]. Other studies in Sweden, the US, Japan, and the Philippines (2010–2016) reported nurses’ insufficient competencies in disaster management [4, 30]. Higher scores of nurses’ competencies in disaster risk management compared with those in previous studies are probably due to their involvement with the COVID-19 pandemic, an increase in the number of training courses for better disaster management, and different data collection tools.

The study results demonstrated that the subscales of technical skills and special diagnostic skills received the highest and lowest mean scores, respectively. Handan et al. in Turkey reported similar results [31]; special diagnostic skills included updating one’s knowledge about specific disasters such as mass casualties and, biological, nuclear, and radiative events. Turkish nurses, like Iranian ones, encountered natural disasters such as floods and earthquakes; the type of disasters where nurses provided care affected their perceptions of competencies. Studies in China [24] and Turkey [29] supported the present study results and reported that technical skills received the highest score, but critical thinking skills received the lowest mean score.

The study results indicated that nurses, who had a master’s degree, participated in training courses related to disaster risk management, and had work experience in previous incidents and disasters, such as earthquakes or road accidents, had higher disaster core competencies. Studies in Indonesia [15] and Poland [32] also reported that continuing education program and academic degrees played important roles in nurses’ preparations and competencies in disaster risk management. A qualitative study showed that nurses had insufficient competencies in disaster risk management due to the lack of knowledge, skills, practical training and necessary exercises; early preparedness in the healthcare system could significantly increase their ability to respond to disaster situations [33].

The study results found that contract recruiters in nursing position with 1–5 years of work experience had fewer competencies in disaster risk management than other groups. The studies conducted in Indonesia and Iran and a systematic review confirmed our results and reported a direct and positive association between work experience, perception, preparation, and skill of disaster risk management [15, 34, 35]. These results indicate that hired nurses perceive occupational security, read the guidelines and programs related to crises and disasters, and use them as standard operating practices or experienced nurses automatically move towards processes that increase their preparations to well manage and lead the care team during disasters.

The study results demonstrated that 43.7% of the nurses had work experience in previous incidents and disasters, such as earthquakes or road accidents, and they reported a high competency score. According to a study conducted in Turkey, 25.4% of the nurses had work experience in previous disasters [29] that had a direct and significant association with the mean score of nurses’ competencies in disaster risk management. Another study in Bangladesh found that nurses with work experience in previous disasters were more competent in responding to disasters than nurses with little or no work experience. Several other studies reported similar findings [36, 37]. Nurses with work experience in disasters are more prepared, have higher skills and competencies in disaster risk management, and they can provide care in disasters even without training and practice.

### Limitations

This descriptive study was conducted to collect information from a self-report questionnaire; therefore, nurses might have increased their actual competency scores. The present study could not investigate causal relationships because it was cross-sectional; experimental studies are recommended to investigate the causal conditions, as well as studies with a larger sample size in other diverse environments with other tools to determine nurses’ competencies in disaster risk management in real situations.

## Conclusion

The study results suggested that nurses’ competencies in disaster risk management were high, with the subscales of technical skills and special diagnostic skills receiving the highest and lowest mean scores, respectively. Managers must take measures to improve nurses’ special diagnostic skills. Nurses with a higher level of education, nurse managers, nurses with work experience in previous accidents and disasters, and those participated in training courses related to disasters better perceived the core competencies required for disaster risk management. Healthcare policymakers and planners should pay more attention, organize, and provide continuous training courses related to disaster risk management for all nurses. Nurse managers should form working groups to use the high capabilities and capacities of nurses at all levels of disaster risk management. One of the effective factors in the disaster preparedness of the hospital and its staff is having a Hospital Disaster Preparedness Plan (HDP) and the level of awareness and participation of the nurses in carrying out that plan.

Therefore, suggested in future studies it would be better to include questions to investigate the knowledge and participation of nurses in running hospital disaster preparedness plans in hospitals.

## Data Availability

The datasets generated and/or analyzed during the current study are not publicly available due to restrictions of the Ethics Committee of Kerman University of Medical Sciences. For available data, please contact: kmu_Research@yahoo.com.

## Abbreviations

NPDCC: Nurses’ perceptions of disaster core competencies scale
HDP: Hospital Disaster Preparedness Plan
COVID-19: Coronavirus disease 2019
START: Simple Triage and Rapid Treatment
SPSS 21: Statistical Package for the Social Sciences 21

## References

[1] Al Harthi M (2020). Challenges for nurses in disaster management: a scoping review. Risk Manage Healthc policy.

[2] Susan A, Joy K. K, *Disaster preparedness need for inclusion in undergraduate nursing education* 2016. 10.18295/squmj.2016.16.01.004 PMC4746037 26909207

[3] Almukhlifi Y, et al. Emergency healthcare workers’ preparedness for disaster management: an integrative review. Journal of clinical nursing; 2021. 10.1111/jocn.15965 34254375

[4] Labrague L (2018). Disaster preparedness among nurses: a systematic review of literature. Int Nurs Rev.

[5] Khankeh HR et al. *A comprehensive review of the articles published in the field of health in emergencies and disasters in Iran*. Pan Afr Med J, 2022. 41. 10.11604/pamj.2022.41.123.31807 PMC9011966 35480412

[6] Songwathana P, Timalsina R (2021). Disaster preparedness among nurses of developing countries: an integrative review. Int Emerg Nurs.

[7] Muhammad-Idris Z, Audu O. *Disasters and Hospital Safety In Nigeria*

[8] Abbasabadi-Arab M, Khankeh HR, Mosadeghrad AM (2022). Disaster risk management in the iranian hospitals: challenges and solutions. J military Med.

[9] Goniewicz K (2021). Cohort research analysis of disaster experience, preparedness, and competency-based training among nurses. PLoS ONE.

[10] Said NB, Chiang VC (2020). The knowledge, skill competencies, and psychological preparedness of nurses for disasters: a systematic review. Int Emerg Nurs.

[11] Huh SS, Kang HY (2019). Effects of an educational program on disaster nursing competency. Public Health Nurs.

[12] Loke AY, Guo C, Molassiotis A (2021). Development of disaster nursing education and training programs in the past 20 years (2000–2019): a systematic review. Nurse Educ Today.

[13] Aliakbari F et al. *Effect of operational exercises on nurses’ competence in dealing with disaster*. J Educ health promotion, 2022. 11. 10.4103/jehp.jehp_429_21 PMC8974978 35372603

[14] Brewer CA (2020). A feasibility study on disaster preparedness in regional and rural emergency departments in New South Wales: nurses self-assessment of knowledge, skills and preparation for disaster management. Australasian Emerg care.

[15] Emaliyawati E (2021). Determinants of nurse preparedness in disaster management: a cross-sectional study among the community health nurses in coastal areas. Open Access Emergency Medicine: OAEM.

[16] Setyawati A-D (2020). Disaster knowledge, skills, and preparedness among nurses in Bengkulu, Indonesia: a descriptive correlational survey study. J Emerg Nurs.

[17] Mizutori M (2020). Reflections on the Sendai Framework for disaster risk reduction: five years since its adoption. Int J Disaster Risk Sci.

[18] Chegini Z (2022). Disaster preparedness and core competencies among emergency nurses: a cross-sectional study. Nurs open.

[19] Choi HS, Lee J-E (2021). Hospital nurses’ willingness to respond in a disaster. JONA: The Journal of Nursing Administration.

[20] Rivera-Rodriguez E. Role of the nurse during disaster preparedness: a systematic literature review and application to public health nurses. Walden University; 2017.

[21] Schumacher L, Bonnabry P, Widmer N (2021). Emergency and disaster preparedness of european hospital pharmacists: a survey. Disaster Med Pub Health Prep.

[22] Ituma OW et al. *Disaster education for australian nursing students: an integrative review of published literature to inform curricula*. Collegian, 2021.

[23] Rizqillah AF, Suna J (2018). Indonesian emergency nurses’ preparedness to respond to disaster: a descriptive survey. Australasian Emerg care.

[24] Fang X-E (2020). Cross-cultural adaptation, validity, and reliability of the chinese version of the Nurses’ perceptions of Disaster Core Competencies Scale (NPDCC). Annals of palliative medicine.

[25] Baker OG (2021). Preparedness assessment for managing disasters among nurses in an international setting: implications for nurses. Int Emerg Nurs.

[26] Kaviani F et al. *Nursing Students’ Competency to Attend Disaster Situations: A Study in Western Iran* Disaster medicine and public health preparedness, 2021: p. 1–5. 10.1017/dmp.2021.263 34802484

[27] Aliakbari F et al. *Development, psychometric testing, and use of a disaster nursing competency scale in Iran: a mixed methods study* Disaster medicine and public health preparedness, 2021: p. 1–6. 10.1017/dmp.2021.197 34399880

[28] Celik F. *Disaster preparedness status of nurses working at Turkish Red Crescent* Unpublished master thesis). Istanbul: Istanbul University Institute of Health Sciences, 2010.

[29] Taskiran G, Baykal U (2019). Nurses’ disaster preparedness and core competencies in Turkey: a descriptive correlational design. Int Nurs Rev.

[30] Goniewicz K (2020). The impact of experience, length of service, and workplace preparedness in physicians’ readiness in the response to disasters. J Clin Med.

[31] Younos TB, Hasan MK, Nasreen M (2021). Are nurses ready? Bangladeshi nurses’ perceived preparedness for disasters: a mixed-methods approach. Int J Disaster Risk Reduct.

[32] Beyramijam M et al. *Disaster preparedness among emergency medical service providers: a systematic review protocol* Emergency medicine international, 2020. 2020. 10.1155/2020/6102940 PMC7683168 33274079

[33] Gardulf A (2019). The Nurse Professional competence (NPC) scale: a tool that can be used in national and international assessments of nursing education programmes. Nordic J Nurs Res.

[34] Hasan MK, Younos TB, Farid ZI (2021). Nurses’ knowledge, skills and preparedness for disaster management of a Megapolis: implications for nursing disaster education. Nurse Educ Today.

[35] Al Thobaity A (2015). Perceptions of knowledge of disaster management among military and civilian nurses in Saudi Arabia. Australasian Emerg Nurs J.

[36] Wenji Z (2015). Chinese nurses’ relief experiences following two earthquakes: implications for disaster education and policy development. Nurse Educ Pract.

[37] Alan H (2022). Nurses’ disaster core competencies and resilience during the COVID-19 pandemic: a cross‐sectional study from Turkey. J Nurs Adm Manag.

